# Supplementary material for: Learning cellular morphology with neural networks
Source: Nat Commun. 2019 Jun 21;10:2736. doi: 10.1038/s41467-019-10836-3 (PMC6588634; doi:10.1038/s41467-019-10836-3)
Supplement: Supplementary file 1 — Supplementary Information [file 41467_2019_10836_MOESM1_ESM.pdf]

Learning cellular morphology with neural networks

Schubert et al.

Supplementary Information

Supplementary Figures

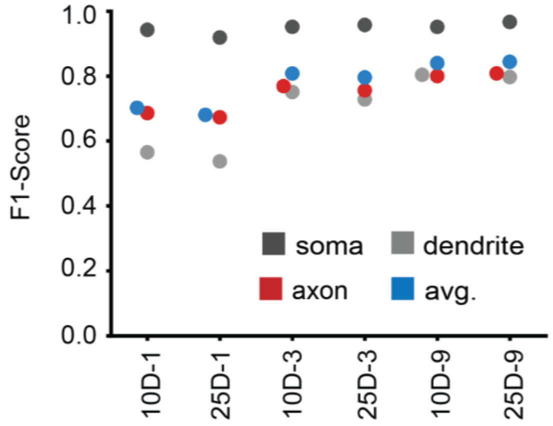

Supplementary Figure 1 Effect of increasing number of nearest rendering locations during similar view sampling on the t-CMN performance with d_z=10, 25 latent dimensions. E.g. 10D-3/9 represents a t-CMN with a 10 dimensional latent space for which the similar view was drawn from one of the two/eight nearest neighbors with p=0.25 during training.

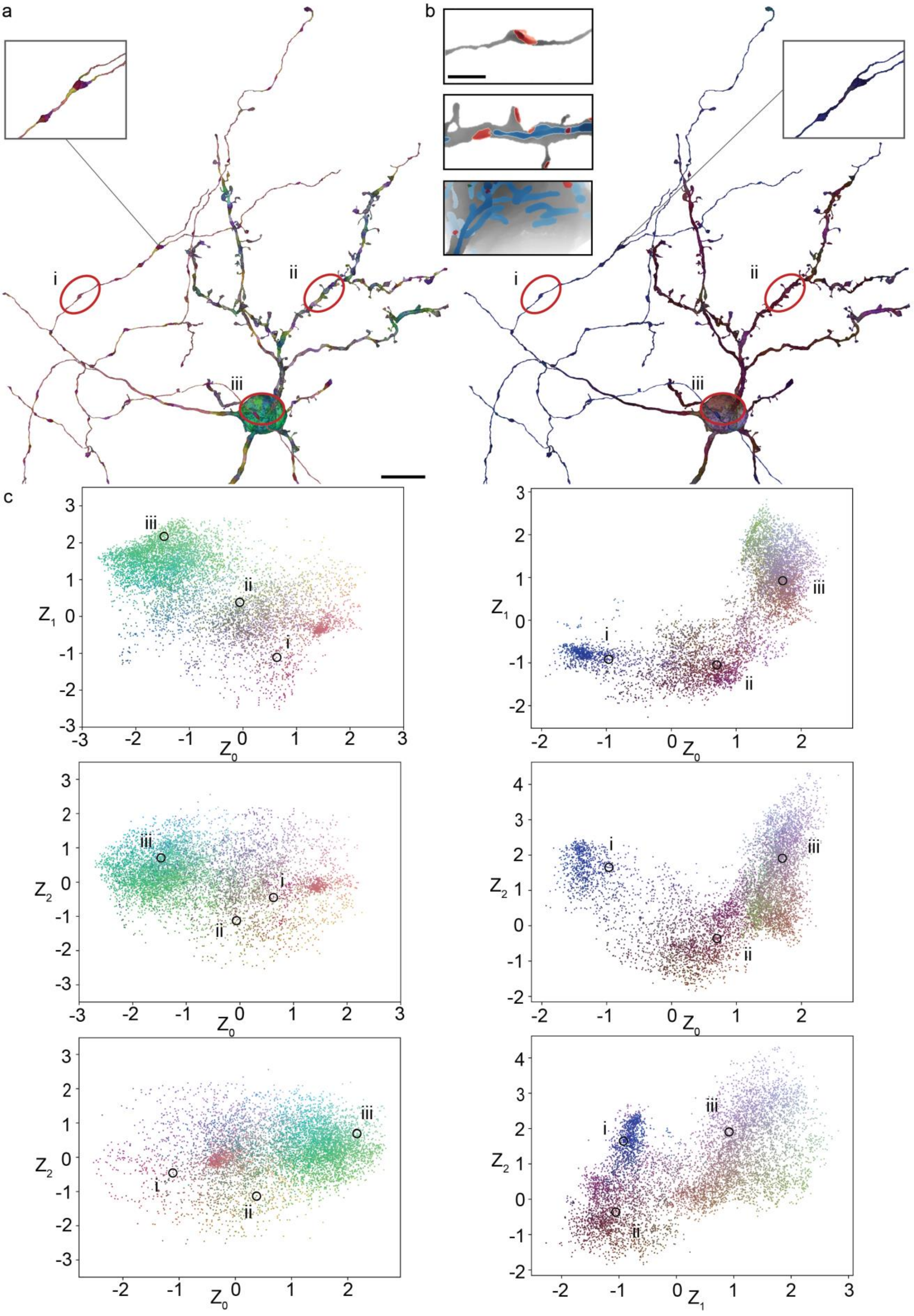

Supplementary Figure 2 Effect of triplet net ground truth adaption on latent space. Left: Latent space dimension d_z=10. Similar view was rotated by 50°. Right: d_z=10. Similar view was rotated by 50° or drawn (p=0.25) from one of the 8 nearest rendering locations. The scale bar in a is 10 µm. **a**

The cell reconstruction renderings were colored according to the top three principal components (captured data variance left: 0.602, right: 0.790). A rendering close-up of one location was added for comparison of the two different latent-colors and to illustrate the smoothing effect. **b** Projection views of the cell and cell organelle surfaces (red: synaptic junction, blue: mitochondria) at the example locations i-iii (from top to bottom, respectively; see **a** and **c**). **c** Principal component distribution of the cell reconstruction. The PCA was fitted to the latent space of the triplet network's training data. Example locations i-iii are indicated in **a** and **c**.

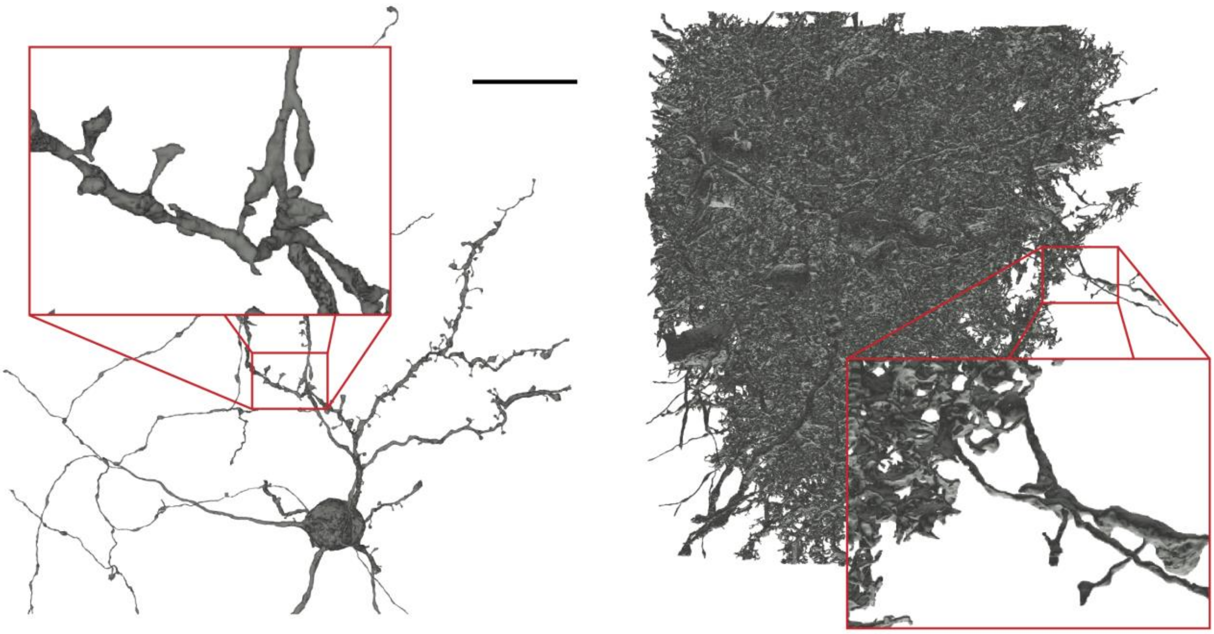

Supplementary Figure 3 Neuron reconstruction (left) and falsely merged neuron supervoxels (right) with glial fragments. Scale bar is 20 µm.

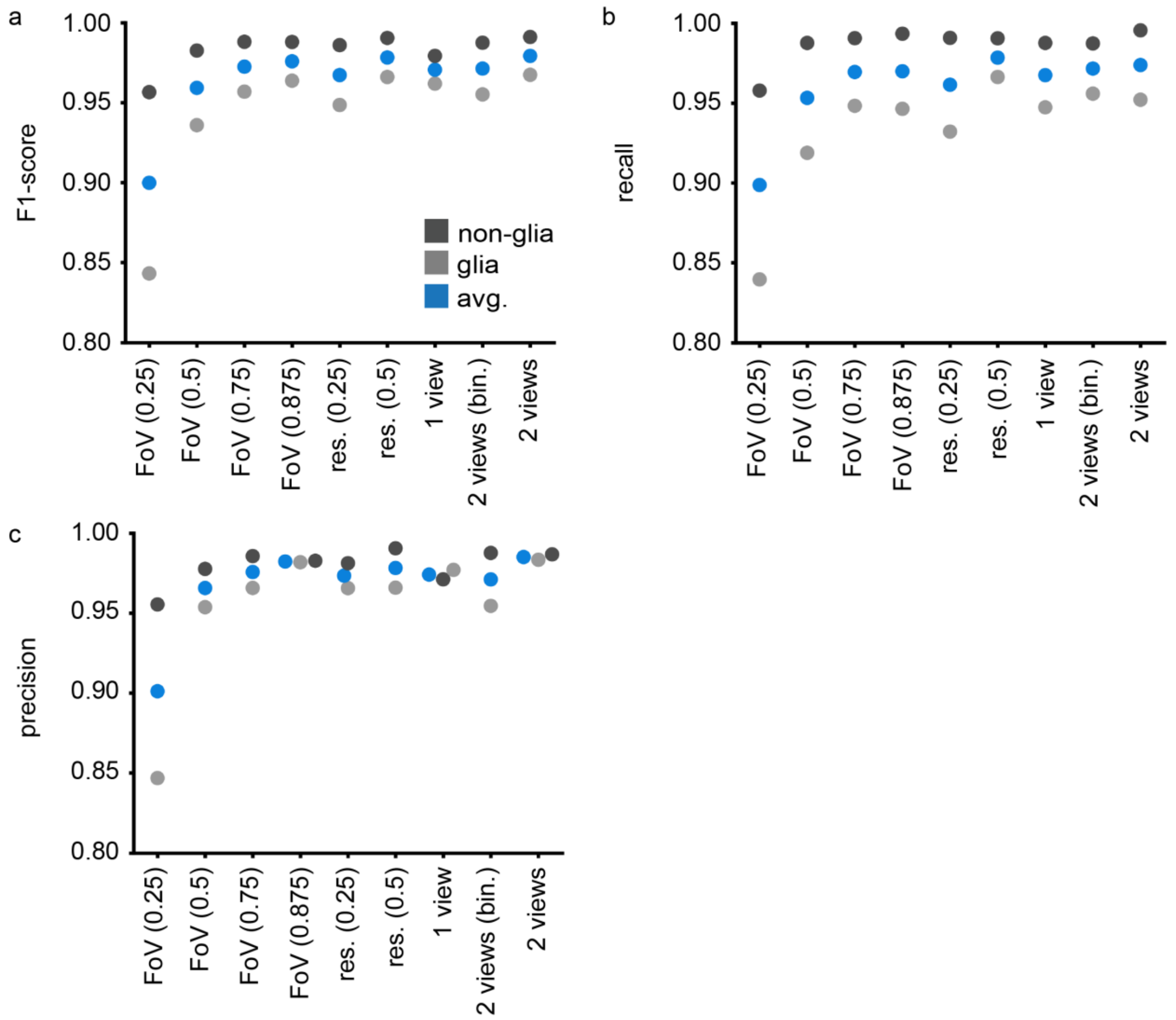

Supplementary Figure 4 Multi-view classification performance on validation set (non-glia: 7,588; glia: 2,107) for different inputs. Left to right: Multi-view resolution was reduced by cropping 3/8 of the images on each side; cropping 1/4 of the images on each side; cropping 1/8 of the images on each side; cropping 1/16 of the images on each side; 4x downsampling; 2x downsampling; single view perpendicular to the 1st and 2nd p.c.; binarized input views; two views at full resolution (256 x 128 px). **a** F1-score. **b** Recall. **c** Precision.

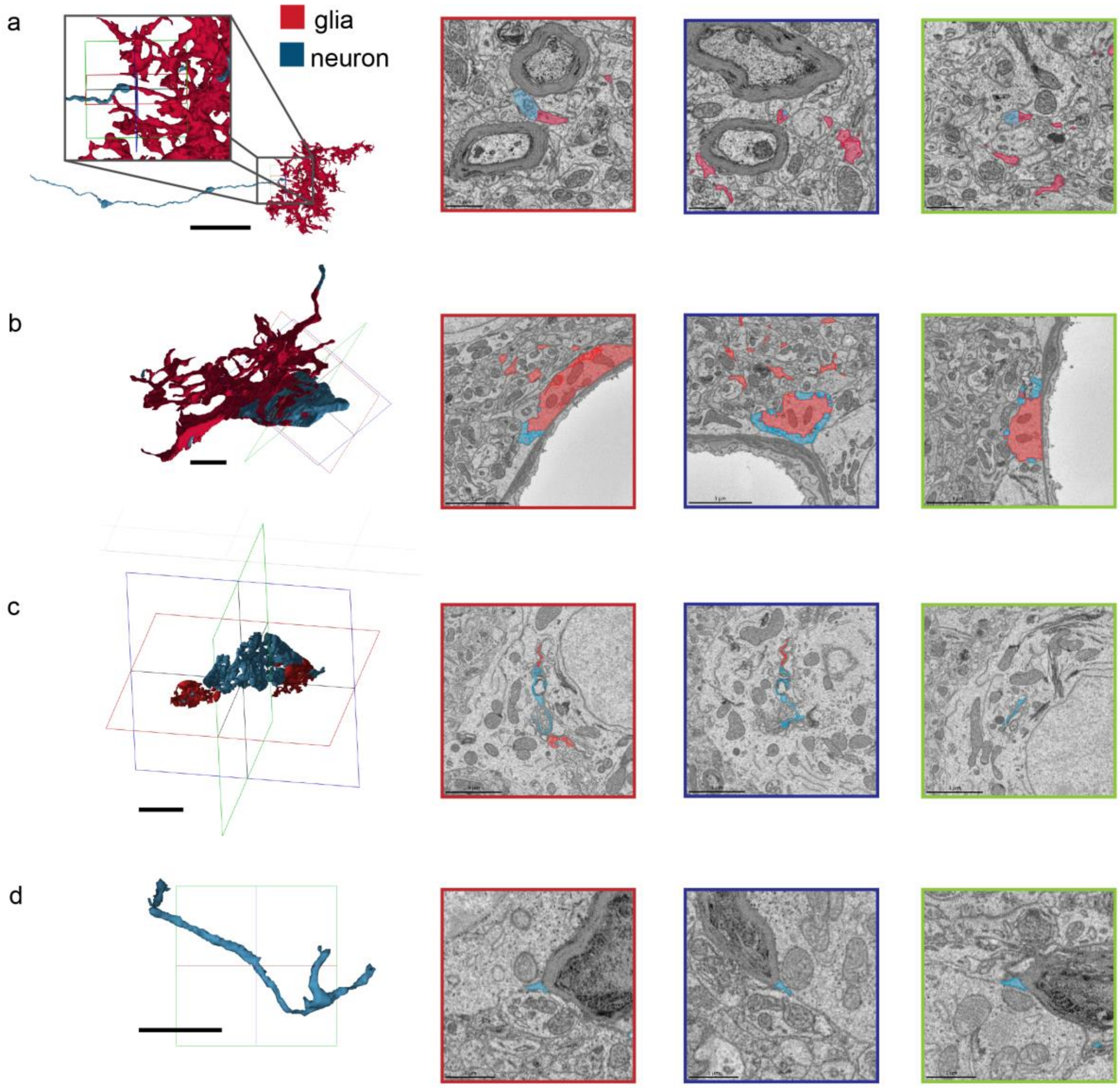

Supplementary Figure 5 Erroneous glia predictions of histogram in Fig. 3b sorted by bounding box diagonal (BBD). **a** Fragments of an axon reconstructions were covered by glia (67 µm). **b** Glial SVs at a blood vessel were predicted as neuron (17 µm). **c** Falsely predicted soma fragments (8 µm). **d** Glial tip fragment touching a myelinated axon was falsely predicted as neuron (8 µm). Scale bars are 10µm in **a** and 2 µm for **b**-**d**.

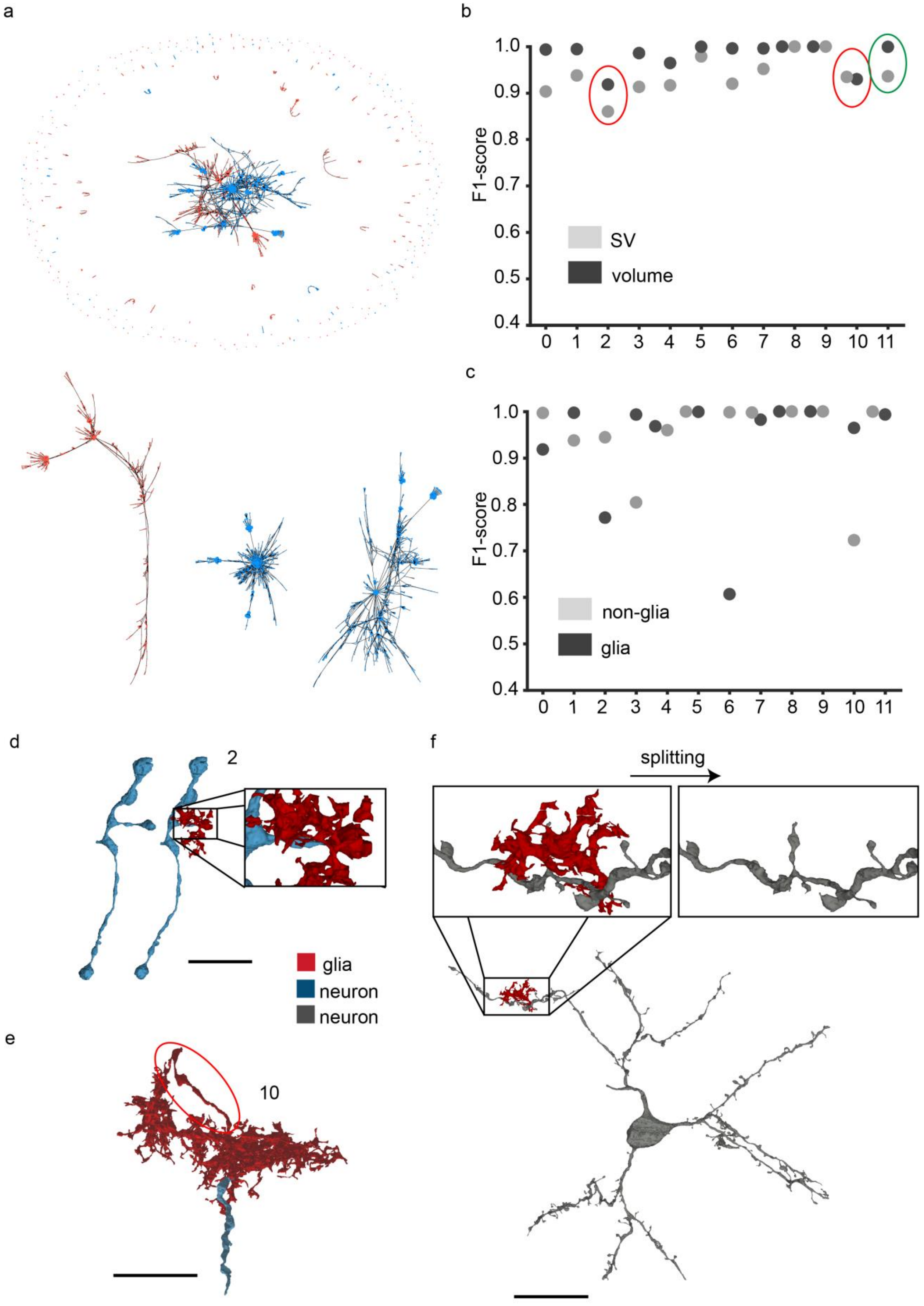

Supplementary Figure 6 Detailed splitting performance values of 12 cell reconstructions and three renderings of split examples. **a** Result of naive splitting procedure (top; removal of edges between glia and neuron SV) vs splitting heuristic (bottom) for reconstruction 11 (see Fig. 3c,d; red: glia; blue:

neuron). **b** Unweighted vs SV-volume-weighted F1-scores of combined non-glia and glia classes (ellipsoids indicate f.l.t.r d, e and Fig. 3d). **c** Non-glia vs glia volume-weighted F1-scores, showing similar error rates for both classes. **d,e,f** Resulting components after splitting procedure: False positive glia prediction example of an axonal terminal bouton (2 in b, c) covered with glia (right) and the actual neuron component (left). Example of a harmful split introduced in an axon-glia merger (10 in b,c). Correctly resolved neuron-glia merger (red: glia; grey: neuron). Scale bars are 10 µm in **d**, **e** and 20 µm in **f**.

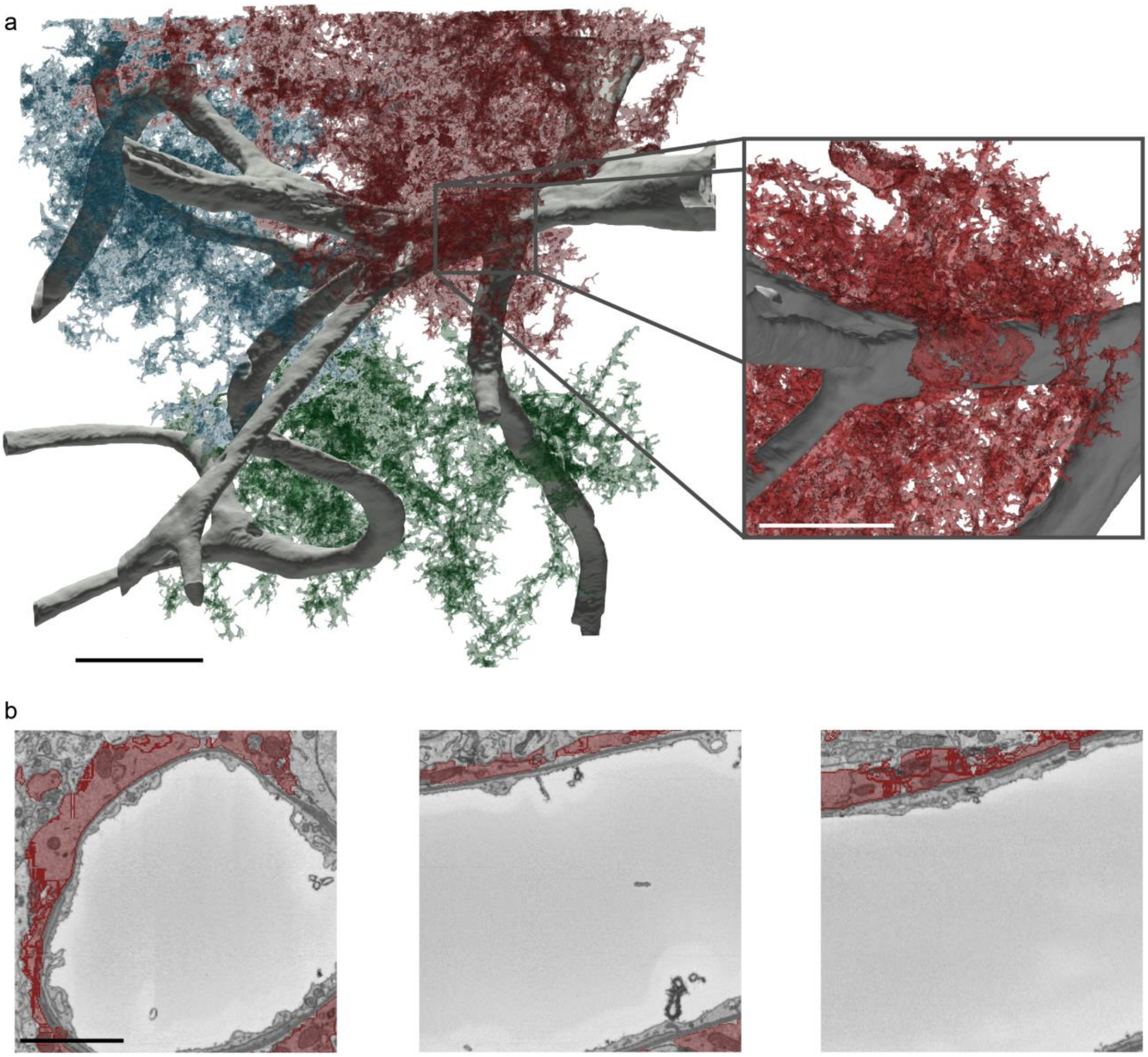

Supplementary Figure 7 Three glial interactions (indicated by color) with a blood vessel **a**. Each color indicates a connected component found by breadth-first-search (BFS) at the contact location. **b** shows the blood brain barrier at the red sample location. The three corresponding EM slices are shown in **b**. Scale bars are 20 µm in **a**, 15 µm for the inset and 3 µm in **b**.

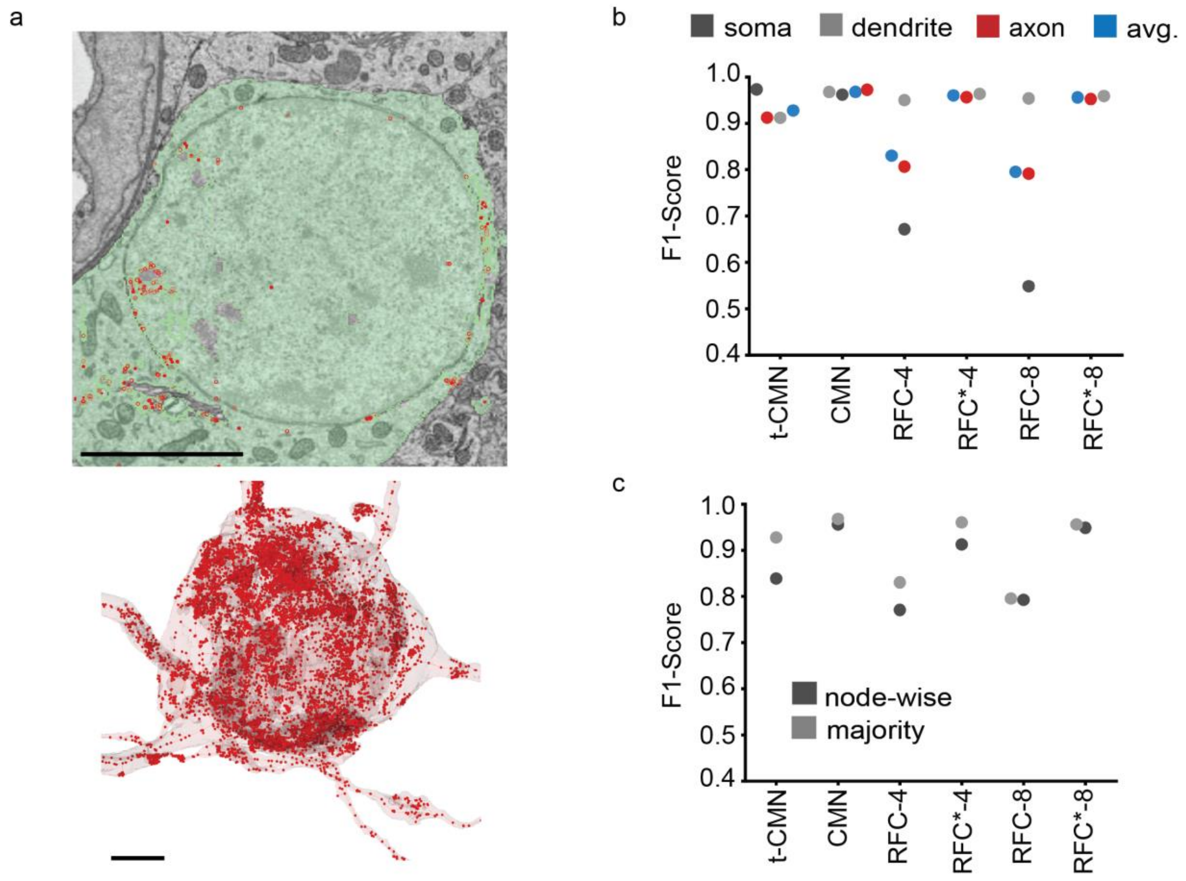

Supplementary Figure 8 Example soma skeleton from and comparison of compartment classification performance on the test set (test set as in Fig. 5c). **a** Example SV segmentation and corresponding skeletonization (red dots and lines) of a soma (top), showing the effect of SV fragmentation on the automatic skeletonization (bottom). **b** Class-wise F1-score after majority vote within sliding windows (see Methods). **c** Comparison of average F1-score between node-wise (Fig. 5c) and sliding window majority classification (b). Scale bars in **a** are 3.5 µm (top) and 2 µm (bottom).

Supplementary Notes

Supplementary Note 1 A vertex-wise performance evaluation (1 MSN dendritic tree reconstruction; 0.27 mm; 0.02 GV; 25 µm^3) showed that the propagation of vertex predictions to uncovered vertices (for coverage ratios see Fig. 6c) using a kNN classifier was only slightly affected by the number of renderings per location and the number of nearest neighbors *k* (F1-score for *k*=1 and 1 view: 0.903; *k*=20 and 6 views: 0.922).

Supplementary Note 2 Manual ground truth painting speeds estimated to be about 12 µm^{3}h^{-1} for mitochondria, 55 µm^{3}h^{-1} for synaptic junctions and 35 µm^{3}h^{-1} based on annotations in a comparable Area X data set (resolution 10 x 10 x 25 nm). Cell type, cell compartment and glia annotations for the training/validation GT took less than 5h using KNOSSOS, since entire SSVs could be labeled, each yielding thousands of view locations. Manual annotation times of node-wise cell compartment (test set) and spine ground truth were 2.3h (2130 µm^3; 933 µm^{3}h^{-1}) and 2.7h (1652 µm^3; 608 µm^{3}h^{-1}), respectively.

Supplementary Tables

step	total time [s]	time / location [s]
render locations (**)	133,14	0,048
PCA rotation	9,67	0,004
cell shape rend. (**)	45,56	0,017
glia pred (**)	45,04	0,016
PCA rotation	9,67	0,004
c.o. rendering (**)	59,40	0,022
comp. prediction (**)	47,99	0,017
c.t. prediction (**)	42,74	0,016
index rendering (**)	191,47	0,070
spine prediction (**)	65,97	0,024
spine mapping	631,63	0,229

Supplementary Table 1 Runtime of each step in the multi-view pipeline. Rows 1-4: glia removal, rows 5-11: neuron analysis. Steps indicated with ** also utilized the GPU. The run time (second column; average of three runs) was measured on two SSVs (854 and 86 SVs; 2447 and 307 rendering locations). Column three shows the average time per location. Abbreviations: Cell organelle (c.o.); cellular compartment (comp.); cell type (c.t.).
